# Fat Therapeutics: The Clinical Capacity of Adipose-Derived Stem Cells and Exosomes for Human Disease and Tissue Regeneration

**DOI:** 10.3389/fphar.2020.00158

**Published:** 2020-03-03

**Authors:** Lipi Shukla, Yinan Yuan, Ramin Shayan, David W. Greening, Tara Karnezis

**Affiliations:** ^1^ O’Brien Institute Department, St Vincent’s Institute for Medical Research, Fitzroy, VIC, Australia; ^2^ Department of Plastic Surgery, St Vincent’s Hospital, Fitzroy, VIC, Australia; ^3^ Plastic, Hand and Faciomaxillary Surgery Unit, Alfred Hospital, Prahran, VIC, Australia; ^4^ Department of Plastic and Reconstructive Surgery, Royal Melbourne Hospital, Parkville, VIC, Australia; ^5^ Molecular Proteomics, Baker Heart and Diabetes Institute, Melbourne, VIC, Australia; ^6^ Department of Biochemistry and Genetics, La Trobe Institute for Molecular Science, La Trobe University, Bundoora, VIC, Australia

**Keywords:** adipose, stem cell, exosome, extracellular vesicles, regeneration

## Abstract

Fat grafting is a well-established surgical technique used in plastic surgery to restore deficient tissue, and more recently, for its putative regenerative properties. Despite more frequent use of fat grafting, however, a scientific understanding of the mechanisms underlying either survival or remedial benefits of grafted fat remain lacking. Clinical use of fat grafts for breast reconstruction in tissues damaged by radiotherapy first provided clues regarding the clinical potential of stem cells to drive tissue regeneration. Healthy fat introduced into irradiated tissues appeared to reverse radiation injury (fibrosis, scarring, contracture and pain) clinically; a phenomenon since validated in several animal studies. In the quest to explain and enhance these therapeutic effects, adipose-derived stem cells (ADSCs) were suggested as playing a key role and techniques to enrich ADSCs in fat, in turn, followed. Stem cells - the body’s rapid response ‘road repair crew’ - are on standby to combat tissue insults. ADSCs may exert influences either by releasing paracrine-signalling factors alone or as cell-free extracellular vesicles (EVs, exosomes). Alternatively, ADSCs may augment vital immune/inflammatory processes; or themselves differentiate into mature adipose cells to provide the ‘building-blocks’ for engineered tissue. Regardless, adipose tissue constitutes an ideal source for mesenchymal stem cells for therapeutic application, due to ease of harvest and processing; and a relative abundance of adipose tissue in most patients. Here, we review the clinical applications of fat grafting, ADSC-enhanced fat graft, fat stem cell therapy; and the latest evolution of EVs and nanoparticles in healing, cancer and neurodegenerative and multiorgan disease.

## Introduction

Adipose dysregulation is fundamental to several important human disease states, such as obesity, chronic lymphedema and lipedema. In contrast to the unwanted effects of excess adipose tissue accumulation, however, adipose tissue also plays a critical physiological role (Fujimoto and Parton, 2011; Rajabzadeh et al., 2019). In humans, fat performs key functions, including energy storage and metabolism, thermoregulation, shock absorption and hormone metabolism (Nishimura et al., 2000; Yoshimura K, 2010). In addition, clinical use of fat tissue has revealed important potential therapeutic applications for adipose tissues in the treatment of human disease (Nishimura et al., 2000; Yoshimura K, 2010). Whilst the clinical use of fat initially began as a physical ‘space filler’ or ‘contour correction’ technique, it was through serendipitous observation of the tissues being filled with fat, that an even more important role has emerged – the role of adipose tissue as a putative therapeutic (Matsumoto et al., 2006).

An adipose derived stem cell (ADSC) is defined as a mesenchymal cell within adipose tissue with multipotent differentiation and self-renewal capacity. Adult stem cells have found an important role in tissue engineering and regenerative medicine, as they may be used to develop novel treatment approaches (Rajabzadeh et al., 2019). In particular, ADSCs are a most promising cell type for translational potential and for cell-based regenerative therapies, as they provide a new and unique source for multipotent stem cells that boasts ease and reproducibility of isolation using minimally invasive techniques with low morbidity. As multipotent ADSCs can differentiate into various cell types of the tri-germ lineages, including osteocytes, adipocytes, neural cells, vascular endothelial cells, cardiomyocytes, pancreatic β-cells, and hepatocytes; the use of fat/ADSCs and their cell products represents a paradigm of tissue regeneration and cell restoration.

Here, we review the treatment of human diseases using adipose tissue from its origins as the humble fat graft, through attempts to enrich the concentration of ADSCs within the grafts; to selective attempts to harness the potential paracrine effects of the ADSC secretome, and finally to the most recent evolution – the targeted use of ADSC exosomes (now known as EVs). We provide a review of the field to date, exploring the therapeutic application of ADSCs and small EVs as delivery vehicles of the ADSC secretome for clinical use in disease. As the focus of the review is ADSC cell products, previous theories of fat differentiating or homing in to replace tissue as ‘building blocks’, are not extensively addressed.

### Fat Grafting, the Stromal Vascular Fraction and ADSCs

#### Fat Embryology, Anatomy and Physiology

Adipocytes that form adipose tissue arise from perivascular adipoblast stem cells in the third month of gestation (Matsumoto et al., 2006) *via* adipocyte precursors, which, in turn, differentiate into mature fat cells (Joseph et al., 2002). After adolescence, minimal new adipocytes are formed, and the role of fat cell replication, is thereafter undertaken by post-adipocytes. The ultimate number of fat cells formed is genetically determined, and slightly influenced by environment and nutrition (Fujimoto and Parton, 2011).

Within adipose tissue, lipid droplets may be uni- or multi-loculated (Fujimoto and Parton, 2011). Unilocular signet-ring shaped fat cells (25-200 µm diameter) are characteristic of ‘white’ fat. Multilocular cells, typically found in so-called ‘brown**’ or ‘beige’** fat, consist of numerous smaller (60 µm) fat droplets (Joseph et al., 2002). Brown fat occurs in smaller quantities near the thymus and in dorsal midline region of the thorax, neck and abdomen (Nueber, 1893; Fujimoto and Parton, 2011) and plays a role in regulating body temperature *via* non-shivering thermogenesis, a mitochondrial mechanism of heat generation *via* a specific carrier called an uncoupling protein (Czerny, 1895; Joseph et al., 2002). In contrast, white fat performs three distinct functions of heat insulation, mechanical cushioning, and an energy source/storage sync; (Illouz, 1986; Joseph et al., 2002). Fat for clinical therapeutic use is sourced predominantly from areas of white fat.

Adipocytes have two different catecholamines receptors (lipolytic β -1 receptors that secrete lipase and α -2 receptors which block lipolysis) (Joseph et al., 2002). During weight gain, fat deposition occurs throughout the subcutaneous and visceral areas relatively evenly (Joseph et al., 2002), into existing adipocytes (hypertrophic growth) (Fujimoto and Parton, 2011). In contrast, when a patient is greater than thirty percent above the ideal weight (body mass index (BMI) over thirty-five), new fat cells are produced (hyperplastic obesity) (Fujimoto and Parton, 2011). Hyperplastic cells are more resistant to dieting and exercise (Tabit et al., 2012). During weight loss, visceral fat is preferential lost, due to greater sensitivity to lipolytic stimulation signals (Joseph et al., 2002). This a process associated with improved insulin resistance (Ross et al., 2014). Bariatric surgery reduces both visceral and subcutaneous fat, leading to overall improved metabolic profiles (Rajabzadeh et al., 2019), however surgery to remove subcutaneous fat (liposuction or abdominoplasty) do not lead to improved metabolic profiles (Ross et al., 2014). The largest amount of visceral fat occurs at level of umbilicus and the greatest amount of subcutaneous fat is found in the region of the buttocks; however, these distributions may vary significantly with gender (Mizuno, 2009). The abdomen and buttocks are the most commonly used areas for fat harvest for fat graft surgery (Ross et al., 2014).

#### The History and Evolution of Fat Grafting

**Figure 1:**
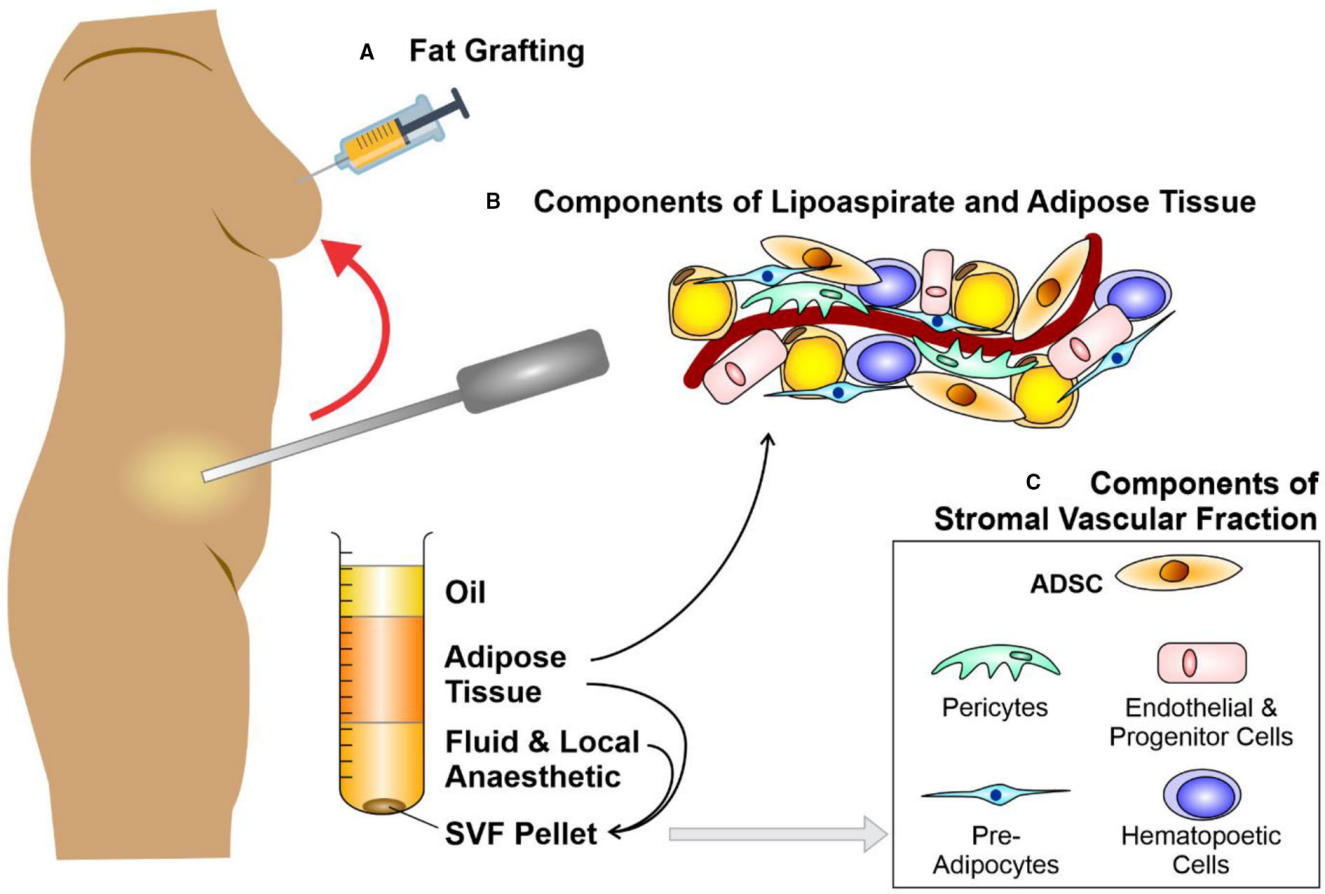
Liposuction, fat grafting and the components of adipose tissue. Schematic diagram depicting **(A)** fat grafting after liposuction of subcutaneous fat from an abdominal donor site. The components of lipoaspirate **(B)** separate into layers of oil (discarded), aspirated adipose tissue and infranatant (composed of blood, plasma, and local anesthetic). The components of adipose tissue and the key constituents of the stromal vascular fraction (SVF) pellet **(C)** may be re-introduced to enhance the fat graft. Further processing of this adipose tissue with collagenase digestion and centrifugation allows the isolation of a SVF pellet. Figure adapted from Shukla et al. (2015) under the CC-BY license (Shukla et al., 2015).

An autologous graft is defined as the transfer of a tissue(s) to a distant area of the body, without its original blood supply (Nishimura et al., 2000) (**Figure 1A**). In order to survive, therefore, a fat graft needs to gain nutrients and a blood supply and from the native tissue bed into which it has been introduced. It needs early revascularization to avoid death of the grafted tissue (Nishimura et al., 2000; Yoshimura K, 2010). Unfortunately, due to poor graft re-vascularization, cell apoptosis or fat cell necrosis, up to 50%–100% of the initial injected volume may fail to engraft and become resorbed (Matsumoto et al., 2006).

Fat graft surgery was first performed by Neuber (1893), then expanded to breast reconstruction when a lipoma (benign fatty lesion) was transferred from the back to reconstruct a breast after cancer surgery (Czerny, 1895). By the 1980s, early rates of graft take [(approximately 50% (Illouz, 1986)] had failed to significantly improved, despite multiple technical refinements in graft harvest, centrifuge or infiltration (Carraway and Mellow, 1990). Irrespective of these loss rates, liposuction techniques using syringe harvest enhanced the popularity of fat grafting for correcting facial contour defects in the 1980s (Rohrich et al., 2004) and led to the introduction of fat grafting for the correction of soft tissue deficits in other body areas (Coleman, 2001; Yoshimura et al., 2008; Tabit et al., 2012; Ross et al., 2014) (**Figures 1A, B**). Nevertheless, significant numbers of patients who underwent fat grafting continued to suffer graft loss, and those in whom graft take was achieved endured up to 70% loss of volume (Matsumoto et al., 2006; Mizuno, 2009).

More recently, fat grafting has been used in staged breast reconstruction following oncological mastectomy, and has been adapted in some settings, to a single-stage, large volume injection procedure (Khouri R, 2009). Various authors have suggested differing methods of injection for achieving optimal graft take, ranging from individual droplet deposits (the so-called pearling technique) to a multilayered and multidirectional lattice configuration as an adaptation to the pre-existing standard 3 mm linear graft injections techniques (Coleman, 2001).

Overall, no consensus had been reached regarding the optimal technical procedure to maximize graft take. Whilst the nuances have been debated, the basic principle is that adipose tissues are removed from beneath the skin *via* minimal-access incisions using a hollow, blunt-ended but perforated steel surgical tube, attached to a source of external suction and collection reservoir.

#### Principles of Fat Grafting, Graft Enhancement and Treatment With ADSCs

Several technical modifications have been described to enhance fat graft reliability. It has been suggested that graft survival occurs through imbibition then angiogenesis (Kilroy et al., 2007) from surrounding tissues, promoted through hypoxic-driven protein growth factors. Therefore, various additions such as collagen, FGF, and insulin (Hong et al., 2010; Baek et al., 2011) were suggested to enhance adipocyte survival; however, did not result in significant graft survival gains. The skin quality overlying areas of fat injection were anecdotally observed to improve, therefore, it was suggested that this may be an influence of stem cells within the introduced adipose cell population (Rigotti et al., 2007). The mesenchymal stem cells (MSCs) thought to be instrumental in these effects were hypothesized to originate from pre-adipocytes (ADSCs) within the stromal vascular fraction (SVF) of liposuction aspirate (Gimble et al., 2011); or from MSCs derived from blood vessels (Eto et al., 2011).

Regardless, of all the different variables in fat grafting, the concept of multipotent stem cells populating fat grafts became the new justification for the use of fat graft. ADSCs became the central focus of enhancing grafts and lately, a potential factor in reversing tissue injury, such as injury occurring in radiotherapy (Haubner et al., 2013). ADSCs were initially isolated nearly 2 decades ago by Zuk and colleagues (Zuk et al., 2001). Eto et al. suggested that ADSCs had lower metabolic demands and were more resistant to the mechanical trauma of fat grafting (Yoshimura et al., 2009; Eto et al., 2012; Trojahn Kolle et al., 2012), and were thus more robust compared to adipocytes (Zuk et al., 2001; von Heimburg et al., 2005; Shoshani et al., 2005; Lu et al., 2009; Tremolada et al., 2010; Suga et al., 2010; Piccinno et al., 2013). Other authors showed enhance graft survival rates due to greater levels of angiogenesis (via either imported endothelial progenitor cells or ADSCs) generating neo-vasculature (Thanik et al., 2009; Zhu et al., 2010; Kolle et al., 2013). Butala et al. on the other hand, postulated that ADSCs in a graft may themselves chemotactically recruit further stem cells, particularly from bone marrow, or differentiate into fat cells themselves (Zhu et al., 2010; Butala et al., 2010; Kolle et al., 2013).

To enhance the abundance of ADSCs within fat grafts (Caplan AI, 2006; Eto et al., 2012; Kolle et al., 2013; Wang et al., 2013) Yoshimura et al. proposed “cell-assisted lipotransfer enrichment” in which the surplus lipoaspirate was separated into components by centrifugation and the lipoaspirate supplemented with additional SVF (Matsumoto et al., 2006; Fraser et al., 2006; Yoshimura et al., 2008; Yoshimura K, 2010; Harfouche and Martin, 2010; Rigotti et al., 2010; Krumboeck et al., 2013). Briefly, SVF [comprised of 10% ADSCs (Zhu Y et al., 2008; Tabit et al., 2012; Akita et al., 2012)] is derived from a lipoaspirate component that is surplus to the volume needed to fill a particular soft-tissue deficit (Ross et al., 2014). Subsequent to digestion with collagenase, centrifugation creates an SVF pellet (**Figure 1C**). Eventually, the SVF pellet is introduced to the lipoaspirate, in readiness for injection with the ADSCs as part of a fat graft (Zuk et al., 2001; Kilroy et al., 2007; Mizuno, 2009; Yoshimura et al., 2009; Yoshimura K, 2010; Tremolada et al., 2010; Trojahn Kolle et al., 2012; Hsiao et al., 2012). A randomized control trial was designed by Kolle et al. to assess enrichment of lipoaspirate with ADSC concentrations of up to 2,000 times over physiological levels (Kim et al., 2009). Quantification using magnetic resonance scans suggested that ADSC-enriched groups yielded higher graft retention volumes (Caplan AI, 2006; Kolle et al., 2013).

Collectively, this work implied that enrichment of fat grafts could increase viability, volume retention, and neo-vascularization, whilst reducing necrosis rates. The findings also supported the theory that adding ADSCs may augment fat graft survival by bolstering adipogenesis, the supporting vasculature and/or diminishing cell apoptosis—key features of the regenerative properties of fat graft (Phinney and Prockop, 2007; Zhu et al., 2010; Collawn et al., 2012; Kolle et al., 2013).

#### Characteristics of ADSCs

ADSCs are defined as plastic-adherent cells (in standard culture conditions) (Dominici et al., 2006; Zimmerlin et al., 2011), cells exhibiting a CD34^+^, CD31^-^, and CD45^-^ cell surface marker profile (Gronthos et al., 2001; Shayan et al., 2006; Yoshimura et al., 2006; Karnoub et al., 2007; Walter et al., 2009; Lin et al., 2010; Zimmerlin et al., 2011; AIHW, A, 2012; Authors on behalf of, I et al., 2012; Baer and Geiger, 2012; Zuk, 2013) and cells showing differentiation multi-potency into mature bone, cartilage, and fat (Zuk PA1 et al., 2002).

In adults, stem cells may uniquely differentiate into more specialized cell types to: i) replenish injured cells, ii) preserve tissue integrity, iii) maintain cell homeostasis during normal growth or healing (Caplan AI, 2006; Kim et al., 2009). Therefore, MSCs show promising utility in tissue regeneration (Ebrahimian et al., 2009; Harfouche and Martin, 2010; Bhang et al., 2011; Yan et al., 2011; Forcheron et al., 2012; Krumboeck et al., 2013; Yuan et al., 2013). As is the case in bone marrow derived MSCs, ADSCs are may undergo differentiation into a variety of distinct mature tissue types including fat, cartilage, bone, skin, muscle, endothelial, and nerve-like cells when grown with a particular set of induction factors (Zuk et al., 2001; Mizuno, 2009; Ebrahimian et al., 2009; Tremolada et al., 2010). ADSCs also boast the additional benefits that the stem cell yield from fat is 500-fold greater than that obtained from bone marrow (Fraser et al., 2006)—[5x10^5^ ADSCs can be derived from 400-600g of fat (Zhu Y et al., 2008; Marigo and Dazzi, 2011)]; and that ADSCs easier and less invasive to harvest overall (Ross et al., 2014; Shukla et al., 2015).

In terms of the cellular secretory profile, ADSCs produce a more extensive range of chemokines, cytokines and protein growth factors (Caplan AI, 2006; Dominici et al., 2006; Kilroy et al., 2007; Locke et al., 2009; Blaber et al., 2012; Carrade et al., 2012; Cawthorn et al., 2012; Hsiao et al., 2012; Strioga et al., 2012). This secretome profile has contributed the understanding that, in contrast to previously held theories that ADSCs would differentiate to actually replace damaged cells (the “building block” or “host replacement” theories (Neuhof and Hirshfeld, 1923; Yoshimura et al., 2006; Kim et al., 2009; Zuk, 2013; Ross et al., 2014); the paracrine effects of the secretome are now considered as more likely to orchestrate the events needed tissue regeneration (Phinney and Prockop, 2007). The distinct makeup of the ADSCs secretome suggested that ADSCs may influence tissue regeneration by altering the biological and molecular cues driving (Gronthos et al., 2001; Kim et al., 2009; Baer and Geiger, 2012; Collawn et al., 2012; Forcheron et al., 2012), angiogenesis (Bhang et al., 2011; Zimmerlin et al., 2011; Matsuda et al., 2013; Yuan et al., 2013) and lymphangiogenesis (Lin et al., 2010; Yan et al., 2011); while suppressing local immune/inflammatory responses (Fraser et al., 2006; Rigotti et al., 2007; Delay et al., 2009; Tremolada et al., 2010; Marigo and Dazzi, 2011; Cawthorn et al., 2012) and reducing fibrogenesis (Tremolada et al., 2010).

Since the time of the initial description of ADSCs, their molecular profile has been the subject of debate (Stone et al., 2003; Mazzola et al., 2011). This has been chiefly due to the description of different ADSC purification and culture protocols and differing use of sub-total vs. whole SVF (Coleman, 2001; Rigotti et al., 2007; Locke et al., 2009; Yoshimura K, 2010; Cawthorn et al., 2012; Strioga et al., 2012).

#### Safety Concerns and Legislative Implementation of Fat Grafting in Clinical Practice

Concerns regarding the use of ADSCs in clinical practice have been three-fold. Firstly, fears arose that introducing stem cells into a former cancer field might encourage recurrent cancer growth due to potential secretion of pro-angiongenic growth factors such as VEGF-A (Ross et al., 2014; Shukla et al., 2015). Secondly, it was hypothesized that chronic calcification occurring in the previously fat grafted areas may make screening/monitoring for the occurrence further cancer difficult (Ross et al., 2014; Shukla et al., 2015). Finally, the addition of components to enhance ADSC efficiency—such as collagenase processing—created the impression that the fat has been significantly altered and therefore ceases to be an autologous tissue transfer, but more a modified therapeutic product (Raposio and Ciliberti, 2017). The first of these reservations was addressed when it was contested that, despite *in-vitro* data that suggesting that introducing stem cells might promote cell proliferation, there was no equivalent definitive evidence *in-vivo* to that effect (Ross et al., 2014; Shukla et al., 2015; Simonacci et al., 2016). The second concern was deemed not to be an issue in the hands of an experienced radiologist, who should be expected to differentiate between benign “post-graft” and suspicious calcification (Ross et al., 2014; Shukla et al., 2015; Simonacci et al., 2016). A recommendation of the American Society of Plastic Surgeons against fat grafting for breast reconstruction was dropped in 2009, and subsequent case studies have upheld an acceptable risk profile. (Ross et al., 2014; Shukla et al., 2015; Simonacci et al., 2016). Finally, the addition of processing to fat graft to enhance take rates has rendered the fat graft unusable in some jurisdictions. In Europe, the use of collagenase digestion in fat grafting is considered to be a significant manipulation of the graft and therefore no longer to be homologous (Raposio and Ciliberti, 2017). The practical use of manipulative steps is therefore likely to remain a restricted procedure, and would likely need to pass regulatory approval steps akin to those stringent steps required of devices or genetically modified cell treatments.

### Functions of ADSCs in Tissue Regeneration

Since the initial observations made in clinical fat grafting, adipogenic differentiation of ADSCs has been thought to result in restoration of tissue contour and volume. Clinical work indicates that there is new fat near the area of the fat graft introduction, which must have occurred *via* either; i) direct differentiation of introduced ADSC into adipocytes; or ii) ADSCs exerting paracrine effects to influence local stem cells to differentiate into adipocytes (Zuk et al., 2001; Rigotti et al., 2007; Delay et al., 2009; Ebrahimian et al., 2009; Kim et al., 2009; Mizuno, 2009; Uysal et al., 2009; Eto et al., 2011; Mazzola et al., 2011; Karathanasis V et al., 2013). The latter has gained favor of late.

#### Differentiation of Transplanted ADSC During Wound Healing

There are several studies demonstrating that transplanted ADSC can potentially promote wound healing by differentiating into specific cell types in animal models of wound healing. For example, Nie et al. showed that intradermally administered ADSCs facilitated wound closure in rats by enhancing re-epithelialization and granulation tissue deposition (Nie et al., 2011). The enhanced wound repair in these rats was attributed to differentiation of ADSC into epithelial and endothelial cells, which accelerated cutaneous regeneration and angiogenesis (Nie et al., 2011). Kim et al. assessed the efficacy of ADSCs in promoting wound healing introduced *via* three different techniques (topical application, intravenous injection and intramuscular injection) (Kim et al., 2019). This study found that mice treated with ADSC exhibited more stratified and differentiated epidermal and dermal layers, with more rapid re-epithelialization and vascularization regardless of the type of ADSC administration compared to control mice (Kim et al., 2019). Further, Wu et al. employed an ADSC-seeded silk fibroin chitosan film in a rat incisional cutaneous wound healing model, and showed accelerated wound healing and colocalization of transplanted ADSCs which displayed enhanced levels of endothelial markers CD31 and alpha-smooth muscle actin (α-SMA) (Wu et al., 2018). These findings were consistent with another study using an acute radiation ulcer model in rats, in which a portion of transplanted ADSCs were also shown to be colocalized with CD31 (Huang et al., 2013). These findings suggest that these ADSCs may have partially differentiated into endothelial cells to promote angiogenesis during wound healing. Lastly, subcutaneously injected ADSCs resulted in a significant increased angiogenesis and enhanced wound healing at 8 weeks post-implantation in rats (Kuo et al., 2016). Unfortunately, however, these studies failed to directly address the question of whether ADSCs promoted wound healing by differentiating into specific cells types, such as epithelial or endothelial cells, or whether—as the authors claimed—that the increased angiogenesis was due to the ADSC secretomes, including VEGF-A (Kuo et al., 2016). A limitation of these studies was that they were conducted using tissue immunofluorescence, which relies on optical co-localization of markers that can be more misleading in terms of positive ADSC and CD31 signals, compared to PCR that will tease out distinct cell populations that co-express numerous specific markers. Finally, no differentiation of ADSCs was detected in a rabbit model of wound healing 7 days after topical application, although the animals treated with ADSCs did increase granular tissue formation in the wound area (Hong et al., 2013). This finding may suggest that the microenvironment in wounds between rodents and rabbits is critically different, or that ADSC differentiation may not play a significant role as the paracrine secretome of the ADSC population. Further research is required to better understand the differentiating capacity of transplanted ADSC *in vivo*.

#### Non-Differentiation Related Mechanisms: Enhancement of Angiogenesis and Lymphangiogenesis

##### Angiogenesis

Injection of the ADSCs into the recipient tissue bed is thought to increase perfusion of injured tissues and/or graft viability by: i) paracrine promotion of angiogenesis, or ii) supporting existing vascular structures. The concepts that support the existence of such regenerative mechanisms are based on several key findings regarding fat grafting in murine ischemic injury models (Eto et al., 2011). These experiments demonstrated that: i) ADSCs may differentiate into CD31^+^ ECs *in-vivo*; ii) there was enhance density of blood vessels and co-localized fluorescent-labeled ADSCs in or near the vessels; and iii) ADSCs formed a vWF^+^ vessel networks in a Matrigel matrix (Karathanasis V et al., 2013). Further, the release of angiogenic growth factors by ADSCs has been shown to promote revascularization and wound healing. These included proteins such as IGF, PDGF-bb, FGF, TGF-β, and interleukins IL-6, IL-8, stromal-related proteins MMP inhibitor 1 precursor, MCP-1, ANG, and SDF-1, and vascular-related proteins such as vascular endothelial growth factor (VEGF) -A, -C, and -D, (Rehman et al., 2004; Benvenuto et al., 2007; Kilroy et al., 2007; Kim et al., 2008; Lu et al., 2008; Ebrahimian et al., 2009; Mizuno, 2009; Pallua et al., 2009; Uysal et al., 2009; Marigo and Dazzi, 2011; Eto et al., 2011; Heo et al., 2011; Zografou et al., 2011; Baer and Geiger, 2012; Forcheron et al., 2012; Hsiao et al., 2012; Kapur and Katz, 2013; Haubner et al., 2013; Jiang et al., 2013; Karathanasis V et al., 2013; Yuan et al., 2013).

##### Lymphangiogenesis

ADSCs secrete lymphangiogenic factors that aid in lymphangiogenesis, improving or reversing lymphedema in damaged tissues. Lymphatic fluid stasis was found to result in increased TGF-β1, exerting a hypothesized further anti-lymphangiogenic effect. Blockade of TGF-β1 and ADSC stimulation, in contrast, lead to increased expression levels within ADSCs of lymphatic endothelial cell markers podoplanin and Prox-1 and of lymphangiogenic growth factor VEGF-C. In addition, the protein growth factors detected in ADSCs that differentiate them from other MSCs (VEGF-D, IGF-1, and IL-8) at baseline, all display pro-lymphangiogenic activity (Ji, 2007; Rigotti et al., 2007; Delay et al., 2009; Avraham et al., 2010; Mazzola et al., 2011; Yan et al., 2011).

#### Anti-Oxidant, Anti-Inflammatory and Anti-Fibrosis Effects

ADSCs may elicit regenerative benefits by exerting anti-oxidant effects, which in turn provide protective effects combatting cellular injury induced by radical oxygen species, hypoxia, and reperfusion effects following ischemia. Protein growth factors that have been implicated include PDGF-AA, HGF, IL-12, G-CSF, GM-CSF, IGFBPs. Pigmented epithelial derived growth factor, Superoxide dismutase may mediate these effects (Chen et al., 2008; Kim et al., 2008; Kim et al., 2009; Heo et al., 2011; Chang et al., 2013). Specific ADSC-induced cytokines have also been shown to modulate immune and inflammatory responses, as BMSCs, and ADSCs restrict the proliferation T-cells and B-cells through NFKB-mediated pathways. Further, IL-6 and IL-8 secretion act as attractants for monocytes and macrophages, which also promote wound healing processes (Ohnishi et al., 2007; Chen et al., 2008; Goh et al., 2010; Heo et al., 2011; Marigo and Dazzi, 2011; Nambu et al., 2011; Forcheron et al., 2012; Rodriguez-Menocal et al., 2012; Kapur and Katz, 2013; Haubner et al., 2013; Jiang et al., 2013).

An additional method of improving epithelialization and wound healing has been shown to be through modulation of granulation tissue formation and of fibrosis. ADSCs co-cultured with fibroblasts *in-vitro* appeared to modify extracellular matrix (ECM) remodeling through down-regulation of gene expression related to production of collagen types I and types III by fibroblasts. Functionally, treatment of keratinocyte and fibroblasts with conditioned media (CM) harvested from ADSC (ADSC^CM^) lead to improved re-epithelialization (Bensidhoum et al., 2005; Francois et al., 2007; Mouiseddine et al., 2007; Ohnishi et al., 2007; Greenberger and Epperly, 2009; Gimble et al., 2010; Goh et al., 2010; Lee et al., 2010; Heo et al., 2011; Nambu et al., 2011; Lee et al., 2012; Rodriguez-Menocal et al., 2012; Zhang et al., 2012; Chang et al., 2013).

Overall, the endogenous stem cell recruitment along a chemokine gradient to the site of injury or inflammation resulted in improved wound healing, truncation of prolonged inflammatory responses and tissue regeneration (Greenberger and Epperly, 2009). Murine models have demonstrated that MSCs respond by aggregating to a site of tissue damage. Studies tracking systemically introduced human MSCs showed that they home to and became grafted into the site of ischemia or of a necrotic injury. In these studies, SDF1α, produced by ADSCs was the key chemoattractant of other stem cells to the injured area of tissue (Bensidhoum et al., 2005; Francois et al., 2007; Mouiseddine et al., 2007; Dewhirst et al., 2008; Greenberger and Epperly, 2009; Gimble et al., 2010; Suga et al., 2010; Eto et al., 2012; Zhang et al., 2012; Chang et al., 2013; Frazier et al., 2013).

#### Implications of Age-Related Changes to Fat Grafting in Clinical Practice

Several clinical applications for adipose-derived stem cell therapy are related to diseases that become more prevalent with age. In studies that examined the changes to the stem cell population, it was found that the differentiation and other functional profiles changes between cells from infanthood, middle age, and elderly donors (Jin et al., 2017). Other studies also demonstrated reduced proliferation and migration profile with age, however, this effect was less marked in adipose-derived cells than it was in bone marrow derived stem cell populations (Efimenko et al., 2015). When stem cells were harvested from aged patients and mice, ADSCs were more robust in terms of potential cell yield than was the case with other MSCs, however, in terms of the paracrine signaling and angiogenic potential of stem cells (e.g., in terms of VEGF-A production), there was a marked impairment seen in cells taken from older donors in both *in vivo* and *in vitro* models (Efimenko et al., 2015). Similarly, clonogenic potential in ADSCs was reduced with age and all the effects were linked to a likely telomere shortening and accumulation of reactive oxygen species-related cellular injury (Efimenko et al., 2015). Overall, aging of donor stem cell populations may form an important limitation of the ability of ADSCs to delivery therapeutic benefits that can be derived from younger donor stem cell populations. This limitation may constitute an indication for ADSC function testing prior to clinical use, bolster the case for procedures to enhance ADSC efficacy, or herald the requirement for a delivery system that by-passes the ADSC itself to harness the paracrine secretome and cell products in a more targeted fashion—such as the use of exosomes.

### Alternative Approaches to Deliver Beneficial Effects of ADSCs: Small Extracellular Vesicles

#### Extracellular Vesicles: Understanding Their Composition

Extracellular vesicles (EVs) are a heterogeneous population of nano- and micro-sized membrane-encapsulated cell particles that are fundamental mediators of intercellular communication. EVs constitute a diverse range of subtypes, namely microvesicles, exosomes, and several other EV populations, classified by The International Society for Extracellular Vesicles (ISEV) (Thery et al., 2018). All cell types continuously secrete EVs to the extracellular environment. EVs contain select proteins, peptides, RNA species (microRNAs, mRNAs, and long noncoding RNAs), lipids, and DNA fragments, that act locally or disseminate through circulation to act at specific distal sites to pleiotropically modulate cellular responses *via* paracrine signaling (Greening et al., 2016; Xu et al., 2018; Rai et al., 2019). The origin, nature, morphology, size and content of EVs are diverse and represent a novel signaling paradigm (Antonyak and Cerione, 2015). EV trafficking has been studied extensively in the area of oncology; however, there is now evidence of their seminal roles in intercellular communication in fetal-maternal signaling (Evans et al., 2019) and metabolism and tissue regeneration - particularly as trafficking intermediates for adipose tissue (Thomou et al., 2017). EVs may be divided into distinct classes, each with differing composition, capacity for selective packaging and potential for targeted delivery (and thus potential roles in disease). Comprehensive examination of the composition and molecular function of EVs in physiology and pathophysiology must be explored in the context of individual cell types, in order to facilitate cell-specific functions and therapeutic use [reviewed in (Greening and Simpson, 2018)].

#### Defining Extracellular Vesicles

Numerous terminologies have been described to define and identify EVs (Gould and Raposo, 2013). Overall, two main classes of EVs exist: large EVs (or shed microvesicles) and small EVs (or exosomes) (Colombo et al., 2014; van Niel et al., 2018). Large EVs (~150–1500 nm) are generated by outward blebbing of specific regions of the plasma membrane (Tricarico et al., 2017; van Niel et al., 2018; Mathieu et al., 2019). Small EVs (30–150 nm) originate as intraluminal vesicles (ILVs) through the endosomal maturation pathway (i.e., multivesicular bodies (MVBs)), which can release ILVs as exosomes into the extracellular space (Raposo and Stoorvogel, 2013).

During their biogenesis, EVs are selectively enriched with diverse cellular bioactive cargo molecules. RNAs (coding, non-coding), DNAs (single-/double-stranded), proteins (peptides, fusion proteins), and lipids are selectively incorporated into distinct types of EVs (van Niel et al., 2018; Mathieu et al., 2019). Further, diverse surface-bound proteins (e.g., receptors, tetraspanins) that are characteristic of the cell of origin, are selectively displayed on secreted EVs and play a crucial role in the recognition of target recipient cells and orchestrating EV localization; as well as uptake by recipient cells (Xu R. et al.,2019).

Although a growing number of studies have investigated the roles of EVs in cell–cell communication, an understanding of specific mechanisms behind their biogenesis and the heterogeneity of EVs and their subtypes remains rudimentary (Greening and Simpson, 2018). The heterogeneity of small EVs and the identification of non-vesicular extracellular content has raised concerns as to the content and function of some exosomes (Jeppesen et al., 2019). Currently, the extent to which small EVs (and exosomes) differ from other EVs in terms of their biogenesis and functions remains ill-defined; and specific markers that distinguish large from small EVs are the subject of much research (Ji et al., 2014; Greening et al., 2017; Greening and Simpson, 2018; Thery et al., 2018; van Niel et al., 2018; Xu et al., 2018; Zhang et al., 2018; Claridge et al., 2019; Jeppesen et al., 2019). This research includes the characterization of EV classes and their subtypes, imaging and tracking of EVs, mechanisms of cell and tissue targeting and internalization, post-translational and transcriptional regulation of EVs and their cargo, and administration and duration (i.e., transient vs. stable) of functional effects (Xu et al., 2016; Greening and Simpson, 2018; van Niel et al., 2018; Xu et al., 2018; Mathieu et al., 2019).

#### Isolating and Purifying Extracellular Vesicles for Biophysical Studies and Clinical Utility

The majority of rapid/one-step approaches for isolating EVs do not account for the fact that samples may contain a mixture of vesicle classes/subtypes and co-isolated contaminants such as high-molecular weight protein oligomers, RNA granules, and protein-RNA complexes (e.g., high-/low-density lipoproteins, argonaute-2/AGO2) complexes (Jeppesen et al., 2019). Varying methodologies for purifying (enriching) EVs and their modified versions exist, including differential (sequential) ultracentrifugation, density-based fractionation, gel permeation chromatography, affinity chromatography using bio-specific reagents (e.g., antibody targets), membrane ultrafiltration using low-centrifugal force, microfluidic devices, and synthetic polymer based precipitation reagents [for a discussion on application, yield/purity and scalability of these methods, see (Xu et al., 2016; Li et al., 2017)]. The choice of which method for EV isolation used depends on the specific research question or proposed use, as outlined below. Further detail of specific guidelines as recommended by ISEV for studies of EVs has been reported elsewhere (Thery et al., 2018).

##### Stringent EV Isolation Procedures

EVs can be isolated and purified depending on the application. For stringent biochemical analysis [e.g. define their luminal cargo—RNA/DNA/lipid/protein species and surface-exposed proteins (Xu R. et al., 2019)] or specific functionality, rigorous purification strategies are critical, including immunoaffinity targeting. Antibody targets that have been successfully employed in this process include those directed against A33 (Mathivanan et al., 2010), EpCAM (Yoo et al., 2012; Tauro et al., 2012), MHC-II antigens (Clayton et al., 2001; Keryer-Bibens et al., 2006), CD45 (Coren et al., 2008; Mercier et al., 2013), CD63 (Caby et al., 2005; Oksvold et al., 2014), CD81 (Oksvold et al., 2014), CD9/CD1b/CD1a/CD14 (Wiley and Gummuluru, 2006), CD24/SWA11 (Rupp et al., 2011), and HER2 (Koga et al., 2005). Further, targeted EV capture based on bio-specific synthetic peptides (Ghosh et al., 2014) and proteoglycan enrichment (Christianson et al., 2013; Balaj et al., 2015) have been described. Other approaches to purify EVs include sequential centrifugal membrane ultrafiltration (Xu et al., 2015) and density-based fractionation using differential centrifugation (i.e., top- or bottom-loaded) (e.g., OptiPrep™/iodixanol) (Ji et al., 2013; Carrasco-Ramirez et al., 2016; Greening et al., 2016; Willms et al., 2016).

##### Generation of EVs for Therapeutic Studies

By virtue of their bioactive cargo EVs have inherent therapeutic potential (Dean et al., 2013; De Toro et al., 2015; Reiner et al., 2017). Small EVs from human MSCs have been used in tissue regenerative medicine to reduce infarction size in a mouse model of myocardial ischemia/re-perfusion injury (Lai et al., 2015). For these studies, large-scale production of functional homogeneous MSC-derived exosomes was accomplished using size-based fractionation. In another therapeutic application, small EVs from dendritic cells (and tumor cells) have been trialed in vaccine studies (Romagnoli et al., 2014; Kunigelis and Graner, 2015; Pitt et al., 2016; Tian and Li, 2017). Navabi et al. described a large-scale production method combining ultrafiltration and sucrose/deuterium oxide for generating good manufacturing (GMP) grade small EVs for use in clinical trials (Navabi et al., 2005).

#### Extracellular Vesicle Regulation of Adipose Function

Several key studies have demonstrated the role of EVs in adipose function. Recently, adipose tissue macrophages were shown to release exosomes containing a specific miRNA to facilitate glucose intolerance (from fat mice population) and insulin resistance (in lean mice population) (Wu et al., 2017). Exosome-containing miR-155 was shown to transfer into insulin target cell types, regulating cellular insulin response, insulin sensitivity, and glucose homeostasis (Wu et al., 2017). The ability of adipose tissue macrophage-derived exosomes to modulate systemic insulin and glucose tolerance *via* different miRNA compositions depended on their adipose phenotype (Wu et al., 2017). Thomou et al. further highlighted the contribution of adipose EVs to adipose function, with 653 miRNAs expressed in serum-derived exosomes from non-obese, or non-diabetic mice (Thomou et al., 2017). Importantly, adipocyte-specific Dicer KO mice were used to deplete adipocyte-derived miRNAs, revealing that exosomes from adipocytes containing miR-99b, inhibited liver FGF21 expression (Thomou et al., 2017). It was further suggested that these changes in FGF21 facilitated the overall phenotype of the Dicer KO mice. Interestingly, Ying et al. demonstrated that such changes were only marginally affected by adipose tissue macrophages-derived exosomes (Wu et al., 2017), indicating that significant differences are present between the miRNA profiles of different cell types within the source adipose tissue. Finally, it was observed that in adipocyte-specific Dicer KO, there was a substantial reduction in circulating exosomal microRNAs (Thomou et al., 2017).

A seminal study by Flaherty et al. identified that adipocytes communicate with adipose tissue macrophages through EVs (Flaherty et al., 2019). This is achieved by directly transferring lipids to differentiate bone marrow precursors into adipose tissue macrophage-like cells, with critical implications for obesity-associated pathologies (Flaherty et al., 2019). The authors highlighted the fact that adipose tissue from lean mice releases ~1% of its lipid content per day *via* exosomes *ex-vivo*, a rate that more than doubles in obese animals. Amose et al. also showed that EVs in human plasma increased significantly with BMI, supporting a role of EVs as metabolic relays in obesity (Amosse et al., 2018). This study demonstrated a key role for large EVs in the transfer of macrophage migration inhibitory factor (MIF) and the link between adipose-derived EVs and macrophage regulation.

Further investigating the role of exosomes in adipose tissue, Crewe et al. showed that adipose tissue EVs modulated crosstalk between adipocytes and stromal vascular cells for metabolic signaling and regulation (Crewe et al., 2018). Quantities of adipose tissue EVs were increased in a fasted state (compared with genetic and diet-induced obesity), partially because of glucagon-stimulated EV secretion from endothelial cells (Crewe et al., 2018). The authors showed dysregulation of important signaling proteins (antioxidant response, mitochondrial respiration) and lipid species involved in stress response. A critical finding was that extracellular molecules are internalized and packaged into EVs (Crewe et al., 2018), representing a new mechanism by which blood-borne signals are integrated into and supplied to adipose tissues.

In addition to influencing fat biology, components of the ADSC secretome have also been shown to promote wound healing and neuro-regeneration, making it an exciting focus for discovery of potential therapeutic targets (Hu et al., 2016; Yim N et al., 2016); particularly as engineering-specific EV delivery systems is now a reality (Yim N et al., 2016).

### ADSCs for Therapeutic Application in Human Disease

Pre-clinical studies of ADSCs and ADSC-exosomes/EVs are listed in **Table 1** and **Table 2**, respectively. As the exosome/EV field is far less advanced than the clinical practice of fat grafting, the respective advances in the clinical application of each are considered together.

**Table 1:** Pre-clinical studies of ADSCs.

Disease model	*In vitro* or *In vivo*	Function	Key findings with ADSC-CM	Reference
Cutaneous wound	*In vitro* and *in vivo*	Wound healing	Reduced UVB-induced wrinkles in mice. Also, ADSC-CM (conditioned media) inhibited UVB-induced apoptosis and enhanced type I collagen synthesis of human dermal fibroblasts	(Kim et al., 2009)
Cutaneous wound	*In vitro*	Wound healing	Accelerated collagen deposits in human dermis through up-regulation of fibroblasts TGF-β1	(Jung et al., 2011)
Cutaneous wound	*In vivo*	Wound healing	Promote neovascularization and wound repair by up-regulating *Tgfb-1*, *Fgfb,* & *Vegf* gene expression	(Hamada et al., 2019)
Cutaneous wound	*In vitro* and *in vivo*	Wound healing	Enhanced neovascularization and re-epithelialization of wounds by up-regulating VEGF, HGF an FGF protein expression	(Nie et al., 2011)
Cutaneous wound	*In vivo*	Wound healing	ADSC + platelet-rich plasma activated Rho GTPase signaling and lead to accelerated wound cell migration & re-epithelialization	(Zhang et al., 2019)
Secondary lymphedema	*In vivo*	Reduce tail swelling	Promote VEGF-C-mediated lymphangiogenesis and anti-inflammatory M2 macrophages recruitment	(Shimizu et al., 2012)
Radiation injury	*In vitro*	Lymph-angiogenesis	Promoted bFGF-mediated lymphangiogenesis in irradiated LECs	(Saijo et al., 2019)
Alzheimer’s disease	*In vivo*	Neurogenesis	Secreted IL-10 and VEGF to reduce Aβ plaques and promote neurogenesis and cognitive functions	(Kim et al., 2012)
Alzheimer’s disease	*In vivo*	Neurogenesis	Reduce oxidative stress and stimulate neuroblast proliferation to improve cognitive function	(Yan et al., 2014)
Parkinson’s disease	*In vivo*	Neuroprotection	Inhibit dopaminergic neuronal cell death and reduce brain mitochondrial damage, restore mitochondrial function	(Choi et al., 2015)
Parkinson’s disease	*In vivo*	Neuroprotection	Improved motor function by increasing BDNF and GFPA	(Berg et al., 2015)
Huntington’s disease	*In vivo*	Neuroprotection	ADSC-extracts improve rotarod test and reduce mHtt aggregates and striatal atrophy *via* CREB-PGC1α	(Im et al., 2013)
Huntington’s disease	*In vivo*	Neuroprotection	Improved rotarod performance and limb clasping, increased survival, protected striatal neurons and decreased mHtt aggregates	(Lee et al., 2009)
Acute kidney injury	*In vivo*	Renal protection	Attenuate I/R-induced renal damage by suppressing apoptosis and inflammation *via* reduction in levels of pro-apoptotic and pro-inflammatory cytokines	(Zhang et al., 2017)
Diabetic nephropathy	*In vivo*	Renal protection	Reduce oxidative stress and inflammation by inhibiting p38 MAPK signaling pathway	(Fang et al., 2012)
Breast cancer	*In vivo*	Tumor promotor or tumor suppressor	ADSC injected into tumor promote tumor growth, c.f. ADSC injected around tumor inhibits tumor growth	(Illouz, 2014)
Breast cancer	*In vivo*	Tumor promotor	Promoted pulmonary metastases by inhibiting miR-20b & activating c-Kit/MAPK-p38/E2F1 signaling	(Xu H. et al., 2019)

**Table 2:** Pre-clincial studies of ADSC-EVs.

Disease model	*In vitro* or *In vivo*	Function	Key findings	Reference
Myocardial I/R injury	*In vivo*	Cardio-protection	Reduced oxidative stress-induced necrosis and apoptosis in myocardium	(Cui et al., 2017)
Acute myocardial infarction	*In vivo*	Cardio-protection	Reduced cardiac apoptosis, fibrosis & inflammation *via* S1P/SK1/S1PR1 pathway & macrophage M2 polarization	(Deng et al., 2019)
Acute myocardial infarction	*In vivo*	Cardio-protection	miR-126-enriched ADSC-exosomes reduced cardiac inflammation & fibrosis, induce microvascular generation & migration	(Luo et al., 2017)
Stroke	*In vivo*	Neuro-protection	miR-126-enriched ADSC-exosomes induced neurogenesis, vasculogenesis & inhibit post-stroke inflammation	(Geng et al., 2019)
Stroke	*In vivo*	Neuro-protection	miR-181-b-5p-enriched ADSC-exosomes promote angiogenesis of brain microvascular ECs post O_2_-glucose deprivation	(Yang et al., 2018)
Neural injury	*In vivo*	Neuro-protection	Reduced neuro-inflammation by suppressing microglia cells activation by inhibiting NF-κβ and MAPK pathways	(Feng et al., 2019)
Neural injury	*In vivo*	Neuro-regeneration	Promote axonal regeneration & myelination in atrophied gastrocnemius by stimulating secretion of neurotrophic factors from Schwann cells	(Chen et al., 2019)
Alzheimer’s disease	*In vitro*	Neuro-protection	Inhibit formation of Aβ plaques and induce neuronal cells proliferation	(Lee et al., 2018)
Huntington’s disease	*In vitro*	Neuro-protection	Reduce mutant Huntingtin protein aggregates, ameliorated abnormal apoptotic protein levels, & restored mitochondrial function	(Lee et al., 2016)
Parkinson’s disease	*In vivo*	Neuro-protection	Reduce gene expression of GFAP, restore astrocytic injury, and increasing dopamine levels	(Meligy et al., 2019)
Acute kidney injury and chronic kidney disease	*In vivo*	Renal protection	Promoted tubular regeneration and inhibit AKI-CKD transition *via* SOX9 activation	(Zhu et al., 2017)
Acute kidney injury	*In vivo*	Renal protection	Combined ADSC + ADSC-exosomes reduce renal inflammation, oxidative stress, apoptosis, fibrosis, & glomerular & tubular damage	(Lin et al., 2016)
Diabetic nephropathy	*In vivo*	Renal protection	Inhibit podocyte apoptosis and induced podocyte autophagy through miR-486-mediated inhibition of Smad1/mTOR signaling pathway	(Jin et al., 2019)
Breast cancer	*In vitro*	Tumor promotor	Promote migration/proliferation of MCF7 human breast cancer cells *via* Wnt/β-catenin signaling pathway	(Lin et al., 2013)
Prostate cancer	*In vitro* &*in vivo*	Tumor suppressor	Inhibit tumor growth by activating caspase-3/7 pro-apoptotic miR-145 pathway	(Takahara et al., 2016)
HCC	*In vivo*	Tumor suppressor	miR-122 enriched ADSC-exosomes increase HCC chemosensitivity & inhibit tumor growth	(Lou et al., 2015)
Breast cancer	*In vivo*	Tumor suppressor	miR-379 enriched ADSC-exosomes inhibited tumor growth over 6 weeks	(O’Brien et al., 2018)

#### Wound Healing

A wound consists of an area of disrupted tissue integrity, architecture and homeostasis. It may be caused by trauma or by thermal or radiation injury (Devalia and Mansfield, 2008; Fry, 2017). The process of wound healing involves a series of organized molecular events including inflammation, neo-vascularization, scar tissue formation, and tissue remodeling (Gurtner et al., 2008); processes tightly regulated by specific growth factors, such as TGF-β, FGF, and PDGF (Grazul-Bilska et al., 2003). In most injuries, wound repair results in scar formation due to recruitment of collagen secreting fibroblasts to enhance the deposition of collagenous ECM (Gurtner et al., 2008). The beneficial effects of ADSC^CM^ on wound healing have been reported in several pre-clinical studies. For example, reduced proliferative capacity and increased apoptosis seen in UVB-irradiated human dermal fibroblasts were reversed with ADSC^CM^ treatment (Kim et al., 2009). Similarly, it was shown that ADSC^CM^ stimulated synthesis of type I collagen by human dermal fibroblasts and reduced UVB-induced wrinkles in mice (Kim et al., 2009). Another study demonstrated that the mRNA expression of types I and III collagens were enhanced in human dermal fibroblasts following treatment with ADSC^CM^ (Jung et al., 2011).

In addition, animal models have shown promising effects of ADSCs on accelerating wound repair. For example, treatment using artificial dermis as a supportive matrix impregnated with autogenic ADSCs in wounded rats resulted in increased vascularization and healing, which was mediated by increased gene expression of genes involved in tissue repair or angiogenesis [e.g., *Tgfb-1* and *-3*, *Fgfb* and *Vegf* (Hamada et al., 2019)]. Also in rats, Nie et al. employed an excisional wound healing model and demonstrated that ADSCs secreted pro-angiogenic mediators both *in vitro* and *in vivo* (e.g., VEGF-A, HGF, and FGF), in-turn promoting neo-vascularization and re-epithelial regeneration of wounds, thus accelerating the wound repair (Nie et al., 2011). Further, the wound healing effects of ADSCs in skin seems to be augmented when administered in combination with platelet-rich plasma containing several different protein growth factors and cytokines, including FGF, TGF-β and PDGF (Zhang et al., 2019). The study suggested enhanced wound closure in treated mice *via* activation of the Rho GTPase signaling pathway, which is involved in cell migration and invasion (Lawson and Ridley, 2018). Collectively, these findings suggest that ADSCs are a potential therapeutic tool for promoting wound healing.

#### Extracellular Vesicles in Wound Healing

Geiger et al. investigated the application of human fibrocyte-derived exosomes in diabetic mice. They found that wound healing was significantly enhanced in all parameters studied (Geiger et al., 2015). Zhang et al. found human umbilical cord MSC-derived EVs to promote re-epithelialization of a wound model and improved the Wnt4 expression profile (Zhang et al., 2015). Similarly, Zhang et al. suggested that MSC-derived exosomes promote collagen formation and angiogenesis (Zhang et al., 2015). ADSC-derived exosome treatment of human dermal fibroblasts seemed to also induce enrichment of the microRNA within the fibroblasts that contribute to healing (Choi et al., 2018). In a murine wound model, Wang et al. suggested that IV administration of ADSC-exosome resulted in reduced scar size and altered metalloproteinases that may improve healing (Wang et al., 2017). Finally, Ren et al. showed that MVs from ADSCs stimulated proliferation and migration of fibroblasts, keratinocytes, and endothelial cells, particularly *via* the AKT and ERK signaling pathways both *in vitro* and *in vivo* (Ren et al., 2019).

#### Radiotherapy Soft Tissue Injury

Radiotherapy (RTX) is administered as part of cancer treatment, either before or after surgery or, unusually, in the absence of surgery (Ross et al., 2014; Shukla et al., 2015). The resulting injury may have devastating consequence in terms of chronic tissue fibrosis and breakdown that may expose vital underlying structures; or can cause secondary pain, contracture and functional impairment. ADSCs have been shown to enhance the quality of skin and soft tissues in clinical RTX injury and in animal models. These influences are thought to be mediated in a paracrine fashion by ADSC-secreted elements that counter the chemokine environment generated by the RTX-injury; this includes anti-inflammatory and anti-apoptotic effects (Ross et al., 2014; Shukla et al., 2015).

Haubner et al. investigated the influences of RTX in blood ECs, and showed enhanced gene expression of pro-inflammatory cytokines IL6, FGF, ICAM-1, and VCAM1. This model of co-culture with ADSCs showed restoration of expression profiles of all RTX-altered cytokines (Haubner et al., 2013). Chang et al. also utilized intra-peritoneal ADSCs after local RTX to show abrogation of inflammation in treatment groups, with restored gastrointestinal tract (GIT) regeneration and enhanced survival (Chang et al., 2013). ADSC treatment was also linked with increased serum levels of IL10, VEGFA, bFGF, and EGF; in addition to increased SDF-1-mediated stem cells recruitment to the injured area (Chang et al., 2013). Further, Kojima et al. and Lim et al. showed protective influences of ADSC against RTX-induced salivary gland irradiation (Kojima et al., 2011; Huang et al., 2013).

In terms of skin and subcutaneous RTX-induced damage, ADSC treatment resulted in improvement in mouse models of chronic RTX-related impaired wound healing and in unwounded RTX-damaged skin [marked by altered collagen-based scar index measurements, increased dermal thickening and reduced fibrosis marker Smad-3 (Sultan et al., 2011; Huang et al., 2013)]. A similar study, investigating ADSC-enriched fat grafting in larger animals exposed to RTX, showed labeled ADSC integration into skin and concomitant enhanced wound repair, epithelialization, subcutaneous fat reserves and lower apoptotic rates. In addition, recruitment and activation of lymphoid cells was seen (Forcheron et al., 2012; Chen et al., 2014).

#### Lymphoedema

Lymphoedema is the chronic swelling of a limb caused by an accumulation of excess interstitial fluid. In time, if unresolved, the fluid accumulation may lead to the formation of excess subcutaneous fibro-adipose tissue (Brorson, 2003). This condition most commonly occurs in a limb and may be the result a developmental malformation that leads to poor interstitial fluid drainage *via* the lymphatic system (primary lymphoedema) (Lee and Villavicencio, 2010). Alternatively, as is the case in most patients, lymphoedema may develop subsequent to a trauma to the lymphatic system. Typically, secondary lymphoedema occurs following surgery or RTX for cancer (in the developed world) or due to filarial infection (in the developing world) that damage lymphatic vessels and impair lymphatic drainage. The pathological features of secondary lymphoedema include inflammation, adipogenesis, and fibrosis.

Shimizu et al. demonstrated the therapeutic potential of ADSCs in lymphangiogenesis by implanting ADSCs into a surgical mouse model of secondary lymphoedema. They showed that ADSCs stimulated lymphangiogenesis by secreting VEGF-C, and enhanced the recruitment of anti-inflammatory M2 macrophages, which were associated with significantly reduced tail swelling in the model (Shimizu et al., 2012). A recent study by Saijo et al. suggested FGF as a novel factor in the ADSC secretome that could potentially contribute to lymphangiogenesis in irradiated human dermal lymphatic endothelial cells (LEC), implying that ADSCs may ameliorate RTX-injury in LECs (Saijo et al., 2019). Counter to this, however, early lymphangiogenesis has been highlighted as a possible risk factor associated with developing the later stages of lymphoedema in a surgical mouse model of secondary lymphoedema; and, paradoxically, pharmacological inhibition of lymphangiogenesis suppressed lymphedema development in the model (Ogata et al., 2016). Thus, whether ADSC-mediated lymphangiogenesis could be therapeutically beneficial in lymphoedema remains elusive and requires further investigation.

Mechanistic and small EV-based functional studies by Greening et al. linked key components of cancer cell-derived EVs to the modulation lymphatic vessel formation and metastasis, demonstrating that lymphatics can also be responsive to secretome components (Carrasco-Ramirez et al., 2016). This study demonstrated critical functional effects on lymphangiogenesis mediated by vesicle surface podoplanin (hitherto considered a passive marker of lymphatic endothelial tissue) on small EVs, using a specific neutralizing monoclonal surface-specific antibody. It also highlighted a key role of podoplanin in biogenesis and release of EVs, and in lymphangiogenesis function. However, the role of the ADSC secretome as a driver of lymphatic repair after RTX or other lymphatic injury, remains to be revealed.

#### Neurodegenerative Diseases

##### ADSCs in the Treatment of Neurodegenerative Diseases

The use of ADSCs has shown promising pre-clinical results in studies investigating several important neurodegenerative disorders, such as Parkinson’s disease, Alzheimer’s disease, and Huntington’s disease. A study using a murine Alzheimer’s disease model showed that treatment with human ADSCs significantly enhanced levels of the anti-inflammatory cytokine IL-10, as well as key neurotrophic (and vasculogenic) factors, including VEGF-A - which led to a marked reduction in Aβ plaques and memory impairment, and elevation of endogenous neurogenesis and dendritic stability (Kim et al., 2012). Furthermore, autologous implantation of mouse ADSCs in mice with Alzheimer’s disease enhanced regeneration of neuroblasts and reduced oxidative stress in the brain, which in turn alleviated cognitive impairment (Yan et al., 2014). Exosomes from ADSCs have also been shown to transfer enzymatically active neprilysin, a Aβ-degrading enzyme, *in vitro* (Katsuda et al., 2013). Importantly, this study showed that ADSC exosome-mediated function was more significant than bone marrow derived MSCs, contributing to prevention of extracellular plaque formation, subsequent pathogenesis and a potential Alzheimer’s disease therapeutic.

In terms of Parkinson’s disease, a common chronic progressive neurodegenerative movement disorder characterized in patients as diminished brain dopamine levels, numerous studies have been performed assessing the therapeutic potential of human ADSCs on a 6-hydroxyldopamine (6-OHDA)-induced mouse Parkinson’s disease model (Berman and Hastings, 1999). Mitochondrial dysfunction in the brain is known to contribute to pathogenesis of the disease by increasing reactive oxygen species and hence oxidative stress, which exacerbates damage to the dopaminergic neurons in Parkinson’s disease (Berman and Hastings, 1999). Choi et al. demonstrated that ADSCs significantly improved behavioral performance by decreasing dopaminergic neuronal cell death and the population of damaged mitochondria in the mouse brain; as well as by recovering mitochondrial functions in the brains of ADSC-injected mice (Choi et al., 2015). It has also been shown that human ADSCs significantly enhanced expression of brain-derived neurotrophic factor (BDNF) and improved motor lost function in the 6-OHDA murine Parkinson’s disease model (Berg et al., 2015), suggesting a pro-healing effect. Interestingly, however, the levels of glial fibrillary acidic protein (GFAP), were shown to be up-regulated in the brain of ADSC-treated animals (Berg et al., 2015). GFAP is a common indicator of dysfunctional astrocytes, the most abundant central nervous system glial cells. They may contribute to the progression of Parkinson’s disease and GFAP upregulation is a possible sign of neuronal regeneration, however, it should be noted that a definitive role for GFAP is not yet agreed upon (Berg et al., 2015).

Huntington’s disease is a progressive, fatal hereditary neurodegenerative disorder characterized by accumulated mutant *Huntingtin* (mHtt) protein in neural cells, which affects mitochondrial energy metabolism to accelerate cell death by progressive brain atrophy. Therefore, altered mitochondrial energy metabolism due to an impaired CREB-PGC1α pathway is a key risk factor in disease progression, which is characterized by an accumulation of mHtt in the brain (Cui et al., 2006; Chaturvedi et al., 2010). Im et al. investigated the influences of cell-free extracts of human ADSC (ASC-E) on R6/2 mice, which developed Huntington’s disease, and found that ASC-E induced activation of the p-CREP-PGC1α pathway and amelioration of mHtt aggregates as well as striatal atrophy in the brain of R6/2 mice (Im et al., 2013). Also, injection of ASC-E in the mouse model slowed progression of the Huntington’s disease phenotype, including weight loss and declining rotarod performance; although the molecular contents of the ASC-E that exerted these therapeutic effects was not assessed in this study (Im et al., 2013). Similarly, ADSC implantation in the R6/2 murine Huntington’s disease model also showed beneficial effects, such as enhanced rotarod performance, limb clasp and survival; and attenuation of striatal neurons loss; as well as diminished brain aggregation of mHtt (Lee et al., 2009). These results were found to be driven by CREB-PGC1α pathway activation (Lee et al., 2009). Altogether, these studies suggest that ADSC treatment could constitute a novel treatment tool useful in ameliorating key pathogenic steps in the development of Huntington’s and other similar neurodegenerative diseases.

#### Exosomes in the Treatment of Neurological Diseases

There have been a few studies demonstrating critical roles of ADSC-exosomes in neuro-protection and neuro-regeneration owing to their capacity to cross the blood-brain barrier (Alvarez-Erviti et al., 2011). For instance, ADSC-exosomes have been shown to mediate functional neuro-regeneration in stroke. Geng et al. demonstrated in a rodent model that miR-126 enriched ADSC-exosomes enhanced neurogenesis and vasculogenesis after stroke (Geng et al., 2019). These results are in keeping with a rat experiment undertaken by Yang and colleagues, in which miR-181b-5p-enriched ADSC-exosomes promoted mobility and angiogenesis of brain microvascular endothelial cells in stroke (Yang et al., 2018). The manner in which exosomes transverse the blood-brain barrier by using transcytosis through endothelial cells are capable of mediating astrocytes to degrade the cell cytoskeleton (Morad et al., 2019), and have only recently been elucidated. Furthermore, neuroinflammation is a major complication of brain injury, which is triggered by the activation of microglia cells in the central nervous system (Dheen et al., 2007). miR-126-enriched ADSC-exosomes were shown to significantly inhibit post-stroke inflammation by suppressing activation of microglial cells and reducing pro-inflammatory cytokine levels in the rat brain (Geng et al., 2019). Feng et al. also demonstrated ADSC-exosomes to inhibit microglial activation by inhibiting the pro-inflammatory MAPK and NF-κβ signaling pathways, which protected rat brain neural cells from injury (Feng et al., 2019).

Potential gene candidates in ADSC-exosomes that underpin these therapeutic effects have been explored using models of neurite outgrowth and sciatic nerve regeneration. Bucan et al. showed rat ADSC-exosomes to contain a range of neurotrophic factors, such as glial-cell derived neurotrophic factor, FGF-1, BDNF, ILGF-1, as well as nerve growth factor (NGF) (Bucan et al., 2019). Schwann cells are also simulated by neurotrophic factors NGF and BDNF and elicited pro-regenerative effects in nerve regeneration after nerve damage (Jessen and Mirsky, 2019). Chen et al. also demonstrated that exosomes derived from human ADSCs enhanced secretion of BDNF and NGF by Schwann cells, which led to increased proliferation, myelination, migration of cells in a dose-dependent manner *in vitro* (Chen et al., 2019). Additionally, this study assessed the effects of ADSC-exosomes on gastrocnemius muscle atrophy (a readout of sciatic nerve injury in rats) and found that treatment with the ADSC-exosome improved muscle atrophy by promoting axonal regeneration and myelination; although exosomal components that exerted these effects remained unidentified (Chen et al., 2019). Lastly, another study showed ADSC-exosomes to inhibit apoptosis and increase proliferation of Schwann cells in rats after nerve injury (Chen et al., 2019); an additional potential mechanism by which the ADSC-exosomes may promote nerve regeneration.

Several other studies demonstrated beneficial effects of ADSC-exosomes on key neurodegenerative diseases. Lee et al. demonstrated that ADSC-EVs significantly reduced the levels of Aβ plaques in Alzheimer’s disease, inhibiting apoptosis of neuronal cells and augmenting neurite outgrowth of neuronal cells *in vitro* (Lee et al., 2018). In Huntington’s disease (Cho et al., 2019), Lee *el al*. showed that Huntington’s disease model that ADSC-EVs profoundly decreased mHtt aggregates and inhibited apoptosis of neuronal cells *in vitro*. Mitochondrial dysfunction was attenuated by activation of the proliferator-activated receptor γ coactivator 1α (PGC1α) and cAMB response element binding protein (CREB)-peroxisome pathways (Cui et al., 2006; Chaturvedi et al., 2010; Lee et al., 2016). Finally, in Parkinson’s disease (McGregor and Nelson, 2019). Meligy et al. studied a rotenone-induced rat model of Parkinson’s disease to demonstrate that ADSC-EVs significantly increased levels dopamine in the treatment group compared to the control (Meligy et al., 2019). In contrast to the overexpression of GFAP seen in animals treated with ADSCs (Clairembault et al., 2014), it was shown that ADSC-EVs markedly *decreased* the gene expression of GFAP, restored astrocytic injury, and improved motor performance in their Parkinson’s disease model (Meligy et al., 2019). This suggested that GFAP may play a different role in neuroprotection in the same model whether treated with ADSCs or ADSC-EVs. Overall, these results indicated that the ADSC-EVs may have reparative potential in incurable neurodegenerative disorders. Further studies are needed to understand the neuroprotective mechanisms by EVs.

#### ADSCs in Renal Diseases

AKI is a complex clinical condition characterized by deteriorating renal function due to decreased renal perfusion, blood supply and glomerular filtration rates, caused by damage to nephron structures (Prowle et al., 2010; Ostermann and Joannidis, 2016). AKI may progress to long-term chronic kidney disease (CKD), for which there is currently no cure (Rafieian-Kopaei, 2013). Thus, prevention of transition of AKI to CKD is critical. Implantation of ADSCs has been shown to yield beneficial effects on rat models of acute kidney injury (AKI). For example, ADSC treatment in an ischemia/reperfusion (I/R)-induced rat model of AKI significantly decreased the number of apoptotic kidney cells and effectively restored urine protein and serum creatinine levels (Zhang et al., 2017). This finding suggested restoration of kidney function by ADSC treatment, and was consistent with the findings by Lin et al. (Lin et al., 2016). Moreover, ADSC treatment lead to markedly reduced expression levels of multiple pro-inflammatory cytokines, for example, IL-6, TNF-α, and IFN-γ; however, was associated with elevated expression of anti-inflammatory cytokine, IL-10 (Zhang et al., 2017) at the mRNA level. Furthermore, ADSC treatment effectively ameliorated diabetic nephropathy by reducing oxidative stress and inflammatory cytokines levels (e.g. IL-6 and TNF-α), by mediating the inhibition of the pro-inflammatory p38 MAPK signaling pathway (Fang et al., 2012), a factor involved in the development of human diabetic nephropathy (Adhikary et al., 2004).

#### Extracellular Vesicles and Renal Disease

ADSC-EVs have been demonstrated to have a pivotal role in protection from the development of AKI. Zhu et al. studied downstream effects of using ADSC-EVs to prevent transition of AKI to CKD, in a mouse model of renal I/R injury. The authors showed that mice treated with ADSC-EVs exhibited decreased renal I/R injury and increased proliferation of renal tubular epithelial cells, thus attenuating AKI (Zhu et al., 2017). Notably, treatment with ADSC-EVs resulted in upregulation of tubular SOX9 gene expression (Zhu et al., 2017), a key gene involved in renal repair and renal tubule epithelial cell regeneration (Kumar et al., 2015; Kang et al., 2016). Furthermore, reduced levels of the pro-fibrotic cytokine TGF- β1 were observed following the ADSC-EV treatment in the model, suggesting that the EVs inhibited TGF-β1-induced renal fibrosis (Zhu et al., 2017), a key feature of CKD (Humphreys, 2018). Another study by Lin et al. demonstrated that inflammation, oxidative stress, apoptosis, fibrosis, and glomerular and renal tubular damage were mitigated by a combined treatment of ADSC-EVs and ADSCs in a rat model of renal I/R injury (Lin et al., 2016).

In diabetic nephropathy, a common variety of CKD due to impaired podocyte autophagy resulting from aberrant activation of the mTOR signaling pathway, a more recent study employed a spontaneous diabetic mouse model to assess the roles of ADSC-EVs (Godel et al., 2011; Tagawa et al., 2016). It was demonstrated that serum creatinine and blood urea nitrogen and total urinary protein levels, indicators of renal dysfunction, were significantly reduced by ADSC-EVs in diabetic mice (Jin et al., 2019). This finding correlated with the study in AKI carried out by Lin et al. (Lin et al., 2016). Additionally, ADSC-EVs were shown to enhance autophagy (the body’s clearance of cellular debris) and diminish podocyte apoptosis by restricting Smad1/mTOR pathway activation *via* miR-486 (Jin et al., 2019). Activation of miR-486 is important as expression of miR-486 has been found to be down-regulated in diabetic patients when compared with non-diabetic individuals (Regmi et al., 2019), implying that miR-486-enriched ADSC-EVs could be a potential therapeutic for treating diabetic nephropathy. Overall, these findings suggest a therapeutic use for ADSCs in kidney diseases such as AKI and diabetic nephropathy, given their capacity to suppress oxidative stress and inflammation; and the possible additional future efficacy of ADSC-EV in AKI.

#### ADSCs in Cancer

A study using a xenograft mouse model of human breast cancer showed that human ADSCs promoted tumor growth when injected *into* a tumor. In contrast, ADSCs inhibited tumor growth when injected *around* the tumor (Illouz, 2014), suggesting distinct influences of ADSCs in different tumor microenvironments. A recent study by Xu et al. showed that ADSCs could promote metastases in mice xenografted with breast carcinoma through ADSC-released stem cell factor-mediated inhibition of miR20b, which in turn, lead to activation of the c-Kit/MAPK-p38/E2F1 signaling pathway and increased expression of HIF-1α and VEGFA (Xu H. et al., 2019). Meanwhile, upregulation of miR20b reduced metastasis of 4T1 breast cancer cells to the lung, suggesting that miR20b acted as a tumor suppressor miRNA, and that ADSCs may be able to induce lung metastases *in vivo*, through miR-20b inhibition (Xu H. et al., 2019). In contrast, miR-20b was also shown to enhance breast cancer proliferation both *in vitro* and *in vivo* by inhibiting expression of the phosphatase and tensin homologue (PTEN) gene (Zhou et al., 2014), a well-known tumor suppressor gene involved in regulation of breast cancer cells (DeGraffenried et al., 2004). This discrepancy may be due to heterogenous roles of miR-20b in regulating breast cancer development in the presence of ADSCs and the ADSC secretome; or may be due to poor study design. Hence, before conclusions can be drawn, this area warrants further detailed studies. Controversies regarding the regulatory approval for use of fat grafting in a former or current tumor bed are summarized above and in (DeGraffenried et al., 2004).

#### ADSC-Derived Extracellular Vesicles in Cancer

Given the capacity of EVs to exert their effects by transferring proteins and RNA to target cells, the effects of EVs in promoting cancer progression has been studied extensively [reviewed in (Xu et al., 2018)]. It appears that ADSC-EVs have dual (or contradictory) functions in regulating tumorigenesis, both by promoting and inhibiting the growth of cancer cells. For instance, platelet-derived growth factors stimulate ADSCs to release EVs containing pro-angiogenic factors—such as Axl (Tanaka and Siemann, 2019), artemin (Banerjee et al., 2012) and stem cell factor (Zhang et al., 2000)—which have been shown to enhance angiogenesis in human microvascular endothelial cells (Lopatina et al., 2014). An *in vitro* study demonstrated that ADSC-EVs promoted migration and proliferation of MCF7 human breast carcinoma cells through activation of Wnt/β-catenin signaling (Lin et al., 2013), although the involvement of angiogenesis was not assessed.

In contrast, there have been a few studies demonstrating that ADSC-EVs can act as tumor suppressors. For example, Takahara et al. demonstrated notable reduction in prostate cancer growth in tumor-bearing mice following ADSC-EVs treatment, an effect mediated *via* activation of the caspase-3/7 pro-apoptotic pathway, itself signaling *via* miR-145 (Takahara et al., 2016). The therapeutic potentials of microRNA-enriched EVs have also been explored in several tumor models. For example, miR-122 is highly expressed in the liver, and loss of miR-122 correlated with development of hepatocellular carcinoma (HCC) in mice (Tsai et al., 2012). Lou et al. demonstrated that miR-122 transfected ADSC-secreted EVs were rich in miR-122, and that uptake of these EVs by cultured HCC cells lead to increased chemosensitivity to chemotherapeutic agents and significant reduction in tumor growth *in vivo* (Lou et al., 2015). Similar results were shown in a breast cancer study (O’Brien et al., 2018) employing ADSC-EVs enriched with miR-379, a tumor suppressor miRNA whose expression is down-regulated in breast cancer (Khan et al., 2013). It was found that the miR-379-enriched ADSC-EVs significantly inhibited tumor growth without adverse effects in mice over the 6 weeks of monitoring (O’Brien et al., 2018). These findings suggested a potential application of genetically engineered ADSCs to promote secretion of EVs encapsulated in tumor suppressor miRNAs may be a promising, novel strategy to treat cancer. However, whether ADSC-EVs have long-term therapeutic effects after withdrawal of administration is unknown.

#### Extracellular Vesicles in Cardiac Disease—Pathology and Cardio-Protection

EVs derived from human ADSCs have been shown to demonstrate cardioprotective roles through their paracrine effects rather than the direct differentiation into cardiomyocytes. Cui et al. used a rodent myocardial I/R injury model to show that ADSC-EVs protected the myocardium from ischemia- or hypoxia- induced necrosis and apoptosis (Cui et al., 2017). Implantation of ADSC-EVs in the rat model resulted in significant reduction in the levels of apoptotic proteins detected (e.g. Bax), and a significant increase in the expression of pro-survival proteins, including Bcl-2 and Cyclin D1 in rat myocardium (Cui et al., 2017). Further, ADSC-EVs exerted cardioprotective effects *via* activation of Wnt/β-catenin signaling (Cui et al., 2017). Another experiment investigating treatment of a rodent model of myocardial infarction with ADSC-EVs profoundly improved cardiac dysfunction by suppressing cardiac apoptosis and fibrosis (Deng et al., 2019). Interestingly, ADSC-EVs promoted macrophage M2 polarization by activating the sphingosine 1-phosphate/sphingosine kinase 1/sphingosine-1-phosphate receptor 1 signaling pathway, which inhibited inflammatory responses and reduced myocardial fibrosis, suggesting that ADSC-EVs may exert potential anti-inflammatory effects (Deng et al., 2019). In addition, Luo et al. employed genetically modified ADSCs to overexpress miR-126 (a microRNA shown to exhibit cardioprotective effects in myocardial infarction) in EVs (Long et al., 2012; Fei et al., 2016). The miR-126-enriched ADSC-EVs significantly decreased myocardial injury by inhibiting inflammation and fibrosis, and enhancing microvascular generation and migration in rats (Luo et al., 2017). Limitations of ADSC treatments for ischemia heart disease include low cardiac retention rates and insufficient concentrations and retained volumes (Li et al., 2019). Numerous clinical trials of ADSCs-derived products have shown promise and an account of completed and ongoing clinical trials using ADSCs are summarized in **Table 3**.

**Table 3:** Completed and ongoing clinical trials of ADSCs.

Diseases	Study phase	Intervention or treatment	Autologous/Heterologous/Allogeneic	Key findings ADSC/EVs	Reference
Fingertip injury	Pilot study	Injections at the site of injury	Autologous	Accelerate wound healing process and recovery of sensory function	(Tarallo et al., 2018)
Idiopathic pulmonary fibrosis	Ib	Intravenous injections of ADSC-derived SVF	Autologous	Similar survival rates disease progression time in untreated populations. Fail to demonstrate any beneficial effect by ADSC therapy	(Ntolios et al., 2018)
Refractory Perianal fistula in Crohn’s disease	III	Local injections of allogenic expanded ADSCs	Autologous	Remission of fistula openings and reduce perianal disease (MRI)	(Philandrianos et al., 2018)
Secondary progressive multiple sclerosis	I/II	Intravenous injections	Autologous	Safe & feasible in patients. No significant changes in safety parameters	(Fernandez et al., 2018)
osteoarthritis	I/IIa	Intra-articular injections	Autologous	Safe and improved pain, function and cartilage volume of knee joint	(Song et al., 2018)
Diseases		Study phase	Intervention or treatment	Autologous/Heterologous/Allogeneic	NCT number
Chronic kidney diseases		I/II	Intravenous injection	Autologous	NCT03939741
Diabetic foot ulcer		I/II	ADSC-enriched fibrin gel	Autologous	NCT03865394
Chronic obstructive pulmonary disease		I	Intravenous injection	Autologous	NCT02161744
Isolated Articular Cartilage Defects		Unknown	ADSC-enriched acellular dermal matrix	Autologous	NCT02090140
Moderate to Severe Chronic Kidney Disease		I/II	Allogenic injection	Allogeneic	NCT02933827
Knee Osteoarthritis		I/II	Intra-articular injection	Allogenic	NCT02784964
Scars or cutis laxa		I/II	Autologous injection combined with laser therapy	Autologous	NCT03887208
Stroke		I	Intravenous injection	Unknown	NCT03570450
Knee osteoarthritis		III	Intra-articular injection	Autologous	NCT03467919
Knee osteoarthritis		Unknown	Transplantation	Autologous	NCT03014401
Vestibulodynia		Unknown	Transplantation	Unknown	NCT03431779
Alopecia		Unknown	Transplantation	Unknown	NCT03427905
Ischemic Heart Disease and Left Ventricular Dysfunction		I	ADSC-enriched VB-C01 collagen patches	Allogeneic	NCT03746938
Facial Rejuvenation		Unknown	Intradermal injection	Autologous	NCT03928444

#### Summary of ADSC-Derived Clinical Trials

The focus of this review is pre-clinical data supporting ADSC-derived therapy; however, it is worth noting that several early clinical trials have been completed. Studies using non-adipose sourced stem cells are not discussed. Trials conducted to assess the benefit of ADSC-derived treatment of wounds, have only reached pilot study or phase I stage in simple cutaneous wounds (Kim et al., 2009; Holm et al., 2018); however, in Crohn’s disease-related peri-anal fistulae, a phase III study (Panes et al., 2018) has shown good efficacy. Similarly, good efficacy has been shown in phase I and IIa studies involving treatment of osteoarthritis (Song et al., 2018) and phase III studies are ongoing at the time of writing (**Table 3**). Finally, promise has also been shown in central nervous system disease [phase I and II studies in multiple sclerosis (Fernandez et al., 2018)].

The dynamic nature of the field warrants close observation of the ongoing results of these clinical studies. It is hoped, however, that the application of genetically modified ADSC-derived small EVs may overcome issues encountered in trials of ADSCs and enhance our capacity to tailor and target future treatment approaches.

## Conclusion

**Figure 2:**
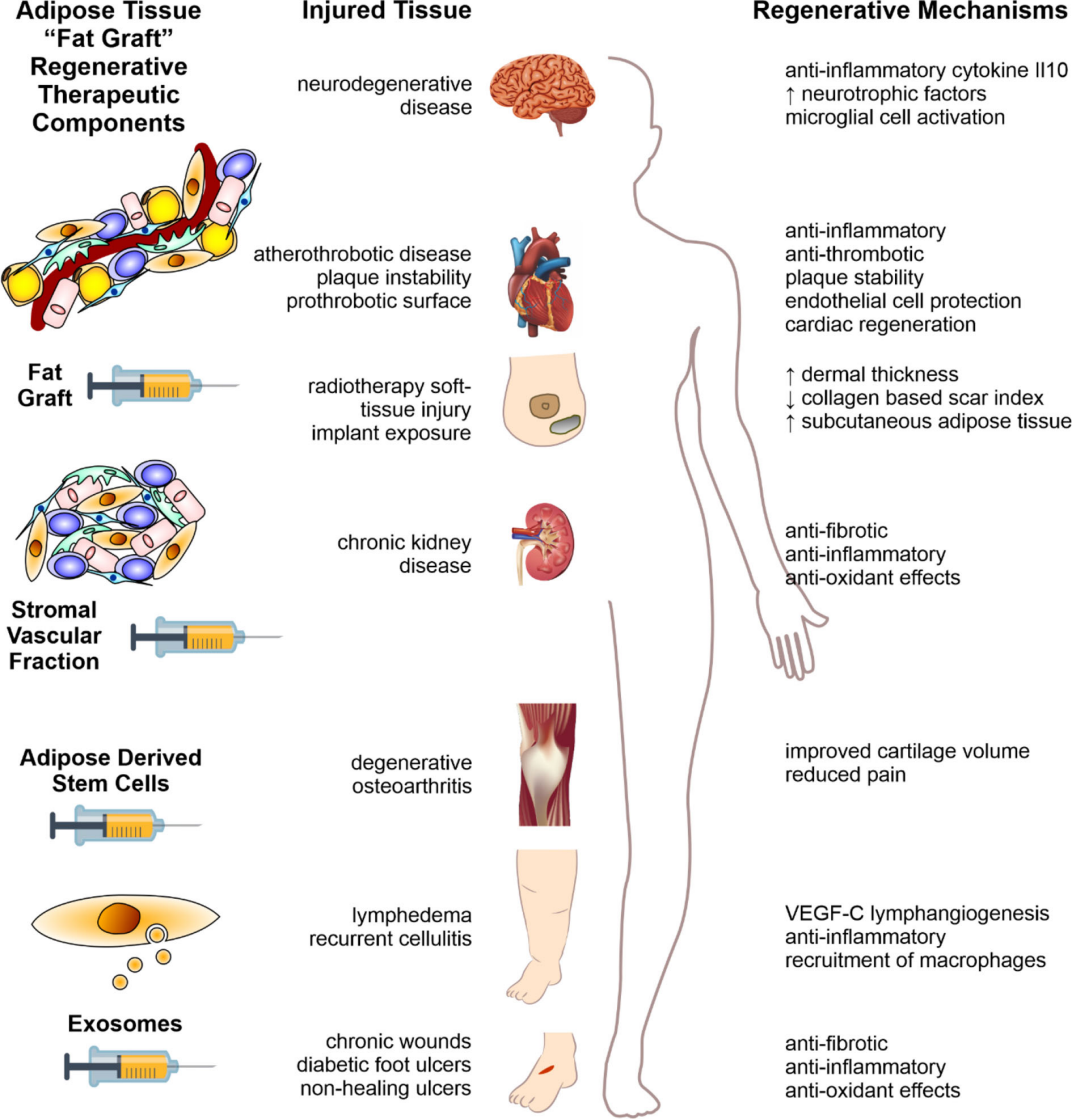
Fat therapeutics of adipose tissue in human disease. Schematic summary of adipose tissue “fat graft” obtained *via* liposuction of subcutaneous fat. Refinement of this fat graft can has occurred at various levels from the acquisition of the rudimentary fat graft, further processed with digestion to obtain the stromal vascular fraction cell pellet, further refinement with extraction of ADSCs, and extracellular vesicle isolation (left column). Each of these components demonstrate significant therapeutic potential in reversing the pathology of human disease, across a range of body systems (middle column). The mechanisms by which these effects are mediated are illustrated in the right-hand column. Figure adapted from Shukla et al. (2015) under the CC-BY license (Shukla et al., 2015).

Fat has played a critical role in basic survival and function throughout the history of human evolution. Now, through evolving the role of fat, humankind may unlock critical answers that assist in novel therapeutic approaches to age-old human diseases; as well as those brought upon ourselves by the evolution of the modern lifestyle. The humble, and until recently rather unfashionable, fat cell may hold the secrets to combatting these diseases—be it through old-fashioned “en-bloc” delivery as raw fat graft, through more sophisticated ADSC-enrichment or cutting-edge discovery and harnessing of paracrine factors in exosomes and other EV types as depicted in **Figure 2**. Together, these insights and the putative treatment that result, may themselves form the cornerstone of the future treatment approaches in regenerative medicine.

## Author Contributions

LS, YY, RS, DG, and TK all contributed to conceptual and figure design, and writing and editing of manuscript. LS and DG contributed to figure creation and YY compiled tables.

## Conflict of Interest

The authors declare that the research was conducted in the absence of any commercial or financial relationships that could be construed as a potential conflict of interest.
